# Genomic flexibility of human endogenous retrovirus type K

**DOI:** 10.1186/1742-4690-10-S1-P29

**Published:** 2013-09-19

**Authors:** Derek Dube, Rafael Contreras-Galindo, Shirley He, Steven King, Marta Gonzalez-Hernandez, Scott Gitlin, Mark Kaplan, David Markovitz

**Affiliations:** 1 Department of Internal Medicine, University of Michigan, Ann Arbor, MI, USA

## Background

Human Endogenous Retrovirus Type K (HML-2), HERV-K, is the most recent group of retroviral proviruses to enter the human genome. In several cases, these HERV-K proviruses have maintained their genomic structure and relevant coding regions relatively unperturbed by the mutations that have inactivated other endogenous viral elements. While no infectious HERV-K virus has been detected to date, resuscitation of infectious clones, derived from consensus sequences of selected HERV-K proviruses, has allowed the historical HERV-K replication cycle to be examined for the first time. Traditionally, it has been thought that HERV-K viruses replicated in a manner similar to canonical retroviruses; that is, with reverse transcription following the entry of an infectious virus particle into a permissive host cell. Here, we provide evidence that, historically, HERV-K viruses may have utilized an additional mechanism for replication, reverse transcribing the RNA genome into DNA prior to viral release from the host cell.

## Materials and methods

HERV-K viral samples were collected from teratocarcinoma cell lines and plasma of patients with lymphoma. HERV-K viral loads were determined by quantitative RT-PCR and PCR, after employing specific DNase and enzyme-treatment procedures to differentiate genomic type (RNA and DNA). Additionally, HERV-KCON [1] and control viruses (MLV and HIV), were produced in the presence or absence of reverse transcriptase inhibitors (RTIs) to examine the infectivity of these viruses.

## Results

HERV-K DNA genomes, in addition to the expected HERV-K RNA genomes, were detected within extracellular HERV-K viral particles from both HERV-K producing cell lines and lymphoma patient plasma, even after the removal of extra-viral contaminating DNAs by DNase-treatment prior to viral lysis. Further, these DNA genomes were determined to represent newly reverse transcribed DNA (RT-DNA), and could be eliminated by culturing the HERV-K producing cell lines in the presence of RTIs. Additionally, using the resuscitated HERV-KCON virus, we found that both HERV-K particles containing RNA genomes and particles containing DNA genomes are capable of infecting target cells. This differs from the canonical retroviruses tested, MLV and HIV, in which infectivity was seen exclusively from viruses with RNA genomes.

## Conclusions

Our data suggest that, both historically and currently, HERV-K virus populations include both RNA and DNA containing particles. This genomic flexibility represents a previously undescribed mechanism of viral replication, and historically would have permitted these viruses to replicate in variable host cell environments, potentially assisting in their many integration events and resulting in their high prevalence within the human genome. Moreover, the ability of modern HERV-K viruses to proceed through reverse transcription and package RT-DNA genomes suggests a higher level of replication competency than previously understood, and may be relevant in current HERV-K-associated diseases.
